# Neural signatures of auditory distraction and cognitive demand during working memory performance

**DOI:** 10.3389/fnins.2026.1919376

**Published:** 2026-09-23

**Authors:** Michael Leitner, Selina C. Wriessnegger

**Affiliations:** Institute of Neural Engineering, Graz University of Technology, Graz, Austria

**Keywords:** cognitive demand, distraction, EEG, neuroadaptive systems, neurophysiology, stress

## Abstract

Cognitive performance in real-world environments often requires maintaining working memory under simultaneous cognitive demand and environmental distraction. Although both factors have independently been associated with alterations in attention and executive control, their combined neural effects remain insufficiently understood. The present study investigated the influence of auditory distraction, time pressure, and their combination on working memory performance using a multidimensional electroencephalography (EEG) framework. Thirty healthy participants completed a working memory task under baseline, distraction, time-pressure, and combined conditions while EEG was recorded from 32 channels. Neural activity was characterized using oscillatory bandpower, topographical statistical mapping, hemispheric asymmetry, and weighted Phase Lag Index functional connectivity. Behavioral performance was complemented by subjective workload and stress assessments. Distraction was primarily characterized by widespread alpha-band suppression, significant frontal and parietal delta-band increases, and decreased frontal alpha asymmetry, whereas time pressure elicited frontal and parietal theta-band power increases. Rather than representing an additive superposition of both manipulations, the combined condition exhibited a distinct spatial pattern of oscillatory changes. Hemispheric asymmetry analyses revealed condition-dependent lateralization patterns that complemented conventional bandpower analyses. While alpha asymmetry primarily differentiated distraction, beta asymmetry emerged as the most consistent hemispheric marker across the experimental conditions showing regional beta-bandpower changes, highlighting the additional value of lateralized EEG measures for characterizing cognitive demands. Functional connectivity analyses did not reveal significant condition-dependent effects. These findings demonstrate distinct oscillatory signatures of distraction, time pressure, and their combination, highlighting the value of a multidimensional EEG framework for investigating concurrent cognitive demands.

## 1 Introduction

### 1.1 Real-world cognitive demands

Humans rarely perform cognitively demanding tasks in isolation. We make decisions while continuously being exposed to competing sensory information, such as music while driving, conversations during learning, visual stimulation in urban environments, or multitasking during digital interaction (Dehais et al., 2020; Li et al., 2023; Hall et al., 2024). Despite these environmental demands, cognitive performance is often maintained through attentional regulation and executive control processes (Miller and Cohen, 2001; Zanto and Gazzaley, 2009; Cavanagh and Frank, 2014). When task demands and auditory distraction occur simultaneously, performance may deteriorate due to overload or overstimulation. Stress and distraction have each been shown to influence attention and working memory (Arnsten, 2009; Sänger et al., 2014; Yan et al., 2024), yet comparatively little is known about how the brain responds when both occur simultaneously. Active cognitive control plays a central role in balancing task-relevant information against competing distractions (Lavie, 2005; Zanto and Gazzaley, 2009; Bonnefond and Jensen, 2012). Previous studies have reported inconsistent findings regarding modality-specific attentional capacity limits for visual and auditory distraction (Guerreiro et al., 2010; Saults and Cowan, 2007), suggesting that the neural mechanisms supporting distraction suppression may differ across sensory modalities. In many real-world situations, cognitive performance must be maintained despite simultaneous stressors and environmental distractions, most of which are processed through visual and auditory modalities. Together with evidence identifying cognitive control as a major performance bottleneck (Cavanagh and Frank, 2014; Baddeley, 2003), these observations motivate a more comprehensive investigation of neural responses. Multidimensional approaches integrating complementary perspectives on local neural activity and large-scale network organization may therefore provide valuable insight into how cognitive resources are allocated and maintained under competing demands.

In the present study, cognitive demand is used as an overarching term for the processing requirements imposed by the working-memory task and its experimental manipulations. Mental workload refers to the subjective effort required to meet these demands and was assessed using the NASA-TLX. Time pressure refers specifically to the experimentally reduced response window. Because the study did not include a condition-specific physiological stress measure, time pressure is treated as a manipulation intended to increase cognitive demand and subjective workload rather than as direct evidence of a comprehensive stress response. Auditory distraction refers to the presentation of task-irrelevant environmental sounds, whereas distractor suppression denotes the cognitive process of limiting interference from those sounds.

### 1.2 Stress and distraction in cognition

So far, established literature has provided direction regarding both the neural networks and analysis levels that may be relevant for stress and distraction. Theoretical frameworks consistently point toward frontoparietal control networks (Baddeley, 2003; Cavanagh and Frank, 2014). Consistent with these frameworks, EEG studies have reported increased interactions between the prefrontal cortex (PFC) and posterior parietal regions during working memory maintenance and attentional processing (Tóth et al., 2012; López et al., 2019). The PFC plays a central role in cognitive control while simultaneously being highly susceptible to stress-related influences (Arnsten, 2009). Consequently, alterations in prefrontal functioning may impair working memory, interference control, and response inhibition (Shields et al., 2016; Baddeley, 2003). These findings establish a direct link between stress exposure and working memory performance, particularly in situations where competing information and attentional distraction place demands on limited cognitive resources.

Consequently, simultaneous exposure to stress and distraction has been demonstrated using electro-physiological approaches revealing alterations in markers associated with attentional allocation and executive processing (Sänger et al., 2014; Gartner et al., 2015; Liu et al., 2020; Zouaoui et al., 2023). A more recent study observed stress-related changes in frontal and frontocentral theta oscillations accompanied by transient improvements in inhibitory control (Yan et al., 2024). They suggest that moderate acute stress can induce adaptive recruitment of executive control mechanisms, highlighting the importance of frontal oscillatory activity during cognitively demanding situations. As cognitive demands increase, attentional bottlenecks emerge and executive systems become progressively constrained by limited processing capacities (Miller and Cohen, 2001; Marois and Ivanoff, 2005). The lateral prefrontal cortex has been identified as a central bottleneck in this process (Marois and Ivanoff, 2005), reflecting its role in coordinating cognitive control across multiple simultaneous demands. Frontoparietal networks are consistently implicated in working memory performance (Tóth et al., 2012; López et al., 2019), supporting attentional allocation, working memory maintenance, and executive regulation. This interpretation is further supported by (Miller and Cohen 2001), who emphasized the limited capacity of executive systems and their susceptibility to overload. Together, these findings provide a direct conceptual link between time pressure, cognitive demand, auditory distraction, and working memory. All four processes rely on overlapping frontoparietal control systems that regulate attentional allocation and cognitive resources. Consequently, simultaneous exposure to stress and distraction may place competing demands on the same neural mechanisms, making their combined influence particularly relevant for understanding the brain's response to increasing task demands.

Distraction competes for limited attentional resources (Lavie, 2005). Within working memory paradigms, distractions interfere with the maintenance of task-relevant information and can impair performance. For example, (Clapp et al. 2010) combined a working-memory task with visual distraction and demonstrated reduced frontal executive control together with enhanced processing in posterior visual regions, reflected in altered event-related responses and blood-oxygenation-level-dependent (BOLD) activity. These changes were associated with impaired working-memory maintenance, indicating that distraction disrupts both executive control and sensory information processing. At the same time, several electroencephalogram (EEG) studies have identified neural mechanisms that support resilience against distraction. In particular, centro-parietal alpha-band increases have repeatedly been linked to the suppression of task-irrelevant information (Bonnefond and Jensen, 2012; Tu et al., 2025; Gutteling et al., 2022). These studies consistently reported increased posterior (parieto-occipital) alpha activity during distraction presentation, suggesting an active shielding mechanism that protects working memory representations from interference. More generally, posterior alpha modulation is widely regarded as a neural marker of attentional allocation and distraction suppression.

When stress and distraction occur simultaneously, maintaining task performance requires efficient cognitive control (Lavie, 2005; Marois and Ivanoff, 2005; Cavanagh and Frank, 2014). Selective attention and working memory rely on shared top-down control mechanisms (Gazzaley and Nobre, 2012), whereby frontal executive systems facilitate the processing of relevant information while suppressing irrelevant sensory input. During working memory tasks containing simultaneous relevant and irrelevant stimuli, frontal control processes have been shown to modulate neural activity in visual sensory cortex through top-down enhancement of relevant information and, more importantly, suppression of irrelevant stimuli (Zanto and Gazzaley, 2009). Greater neural suppression of distracting information was associated with better working memory performance, highlighting the importance of top-down regulation. Within this framework, frontal theta oscillations are associated with cognitive control (Cavanagh and Frank, 2014), whereas alpha-band modulation reflects information filtering and attentional maintenance (Bonnefond and Jensen, 2012; Gutteling et al., 2022). Consequently, oscillatory activity within frontal and parietal regions provides a suitable neural marker for investigating top-down control mechanisms under simultaneous cognitive demands (Sadaghiani and Kleinschmidt, 2016).

Real-world cognition rarely involves only a single source of cognitive demand. As emphasized by (Dehais et al. 2020), previous work suggests that stress and distraction may interact in realistic environments. Successful performance therefore is thought to involve cognitive regulatory processes that remains effective despite competing demands. A paradigm combining simultaneous stress and distraction represents an important step toward a more ecologically valid assessment of neural processes. Furthermore, previous work suggests that stress and distraction may interact and compete for limited cognitive resources (Sänger et al., 2014; Marzolla et al., 2024). Understanding these relationships may also contribute to the development of neuro-adaptive technologies. Working memory tasks provide a particularly suitable framework for studying cognitive demand because they require continuous attentional maintenance while remaining highly sensitive to stress-related and interference-related manipulations. Moreover, the underlying EEG patterns have already been partially characterized, making them promising candidates for investigation within a multi-stimulus paradigm.

### 1.3 Multidimensional EEG analysis

The present study investigates the neural effects of distraction, time pressure, and their simultaneous occurrence during working memory performance using a multidimensional EEG approach. By integrating oscillatory bandpower analysis, hemispheric asymmetry measures, topographical statistical mapping, and weighted Phase Lag Index (wPLI) connectivity analysis (Vinck et al., 2011), the study aims to characterize both distinct and overlapping neural responses associated with increasing cognitive demands. Bandpower characterizes regional oscillatory spectral activity (Klimesch, 1999), functional connectivity analyses provide complementary information about functional interactions between distributed brain regions (Sadaghiani and Kleinschmidt, 2016; Kamzanova et al., 2020), and hemispheric asymmetry captures lateralized processing strategies (Ocklenburg et al., 2019). To statistically identify and localize spatial differences in oscillatory activity between experimental conditions, electrode-wise linear mixed-effects models (LMEs) were additionally employed. The joint evaluation of these complementary analytical approaches provides a comprehensive perspective on cognitive demand that cannot be obtained from any single analytical domain alone.

Electrode-wise LMEs have previously been applied to identify statistically significant spatial EEG modulations (Michel and Murray, 2012; Wriessnegger et al., 2024, 2025). Within this framework, t-statistics derived from LME models are projected onto the scalp, enabling a topographical representation of statistically significant condition-related neural activity. This approach facilitates the identification of spatial recruitment profiles and provides an intuitive visualization of statistically robust effects.

### 1.4 Gap in current research

Although both stress and distraction have been extensively investigated in the context of working memory, these factors are typically studied in isolation. Consequently, it remains unclear how simultaneous exposure to stress and distraction influences neural processing and whether their combined effects represent a simple superposition of established EEG patterns or a reorganization of cognitive control mechanisms. Addressing this question is particularly relevant because both factors place demands on overlapping attentional and executive control systems and frequently co-occur in real-world environments.

Recent studies have examined individual aspects of this problem. For example, (Hall et al. 2024) investigated distraction during working memory performance and reported bilateral prefrontal oscillatory changes, whereas (Yan et al. 2024) focused on stress-related modulation of cognitive control and frontal theta activity. Similarly, (Sänger et al. 2014) observed significant cortisol and alpha-amylase increases as well as increased error rates during a socially evaluated cold pressor test. They provided evidence for acute stress-related alterations in attentional processing, but did not employ a paradigm containing simultaneous competing demands. More recently, (Marzolla et al. 2024) introduced a combined stress-distraction paradigm, demonstrating the relevance of studying multiple cognitive demands concurrently. However, no physiological or neural measures were acquired, leaving the underlying neural mechanisms largely unexplored. Beyond the experimental paradigm itself, a second limitation concerns the level of neural analysis. Many EEG studies focus on isolated neural markers, such as frontal theta increases associated with cognitive control (Cavanagh and Frank, 2014), parieto-occipital alpha-band modulation related to upcoming distractors (Bonnefond and Jensen, 2012), or event-related potentials (Li et al., 2023). While these approaches have provided important insights into neural processing, they often characterize only local aspects of brain activity.

In contrast, cognitive control is increasingly understood as an emergent property of distributed frontoparietal networks, suggesting that network-level measures should be considered alongside local oscillatory activity. Functional connectivity has become an important approach for investigating large-scale neural coordination during cognitive processing (Siegel et al., 2012; Bastos and Schoffelen, 2016; Sadaghiani and Kleinschmidt, 2016). Previous work demonstrated increased frontoparietal theta synchronization during working memory processing and changed alpha oscillations without a global increase or decrease (Dai et al., 2017; Muthukrishnan et al., 2020). Nevertheless, studies integrating oscillatory activity and functional connectivity within a unified framework remain scarce. More broadly, multidimensional EEG approaches that simultaneously investigate local bandpower, large-scale network organization, and hemispheric specialization are still relatively uncommon despite their potential to provide a more comprehensive understanding of neural responses (Babaeeghazvini et al., 2021).

To address these limitations, the present study investigates auditory distraction, time pressure, and their simultaneous occurrence during working memory performance using a multidimensional EEG framework. Oscillatory bandpower, hemispheric asymmetry, topographical statistical mapping, and functional connectivity are analyzed jointly to characterize both local and network-level neural responses. Beyond advancing the understanding of cognitive demands, such multidimensional approaches may contribute to future neuro-adaptive technologies (NATs) in domains such as driver assistance, aviation, adaptive learning, and workload monitoring in medical or safety-critical environments, where distinguishing adaptive regulation from emerging overload states remains a critical challenge (Dehais et al., 2020; Dai et al., 2017).

### 1.5 Hypothesis

It was hypothesized that auditory distraction and time pressure would differentially modulate oscillatory activity and functional brain organization. Specifically, increased frontoparietal theta activity and connectivity were expected under time pressure, reflecting enhanced cognitive control and executive coordination. In contrast, auditory distraction was expected to induce frontal alpha-band modulation associated with attentional allocation, information filtering, and distractor suppression. Especially during distraction, globally decreased oscillatory alpha bandpower was expected. Based on previous findings linking slow oscillatory activity to working memory maintenance and attentional processing, delta-band activity was additionally expected to show condition-dependent modulation, although no strong directional hypothesis was formulated because of the comparatively limited and less consistent literature. Furthermore, hemispheric asymmetry and topographical activity patterns were expected to differ across conditions. Specifically, stronger right-lateralized alpha suppression was expected under increased cognitive demands and time pressure, whereas beta asymmetry was examined to characterize potential lateralized changes in top-down processing. In addition, increased beta-band activity was expected over right frontal and parietal regions, consistent with previous reports relating beta oscillations to top-down cognitive processing. Finally, the simultaneous occurrence of distraction and time pressure was expected to induce the most pronounced changes of oscillatory and network-level activity compared to the baseline condition. Rather than representing a simple superposition of isolated effects, the combined condition was hypothesized to exhibit a distinct pattern of oscillatory and network-level measures, reflecting increased demands on cognition.

## 2 Methods

### 2.1 Paradigm

The experimental design was inspired by modulations from previous paradigms described by (Wriessnegger et al. 2024, 2025). The complete procedure is illustrated in Figure 1. Following written informed consent and EEG preparation, the experiment began with a 15 s eyes-open resting-state recording. Participants then completed 15 calibration trials, followed by the first PASA questionnaire (Gaab, 2009) and NASA-TLX assessment (Hart and Staveland, 1988) were completed directly on screen. The experimental phase followed a block design comprising four conditions, each repeated four times. Every block consisted of 30 trials, resulting in a total of 16 task blocks and 480 trials per participant. To reduce order effects, the starting condition was varied across the four condition quartets while preserving the overall block structure. The complete timeline and condition sequence are shown in Figure 1. Trials within each block were pseudo-randomized. Each task block was followed by a 15 s eyes-open resting-state recording. After completion of all 16 blocks, participants again completed the PASA and NASA-TLX questionnaires before the experiment ended. Finally, all participants were debriefed regarding the experimental manipulations and performance feedback.

**Figure 1:**
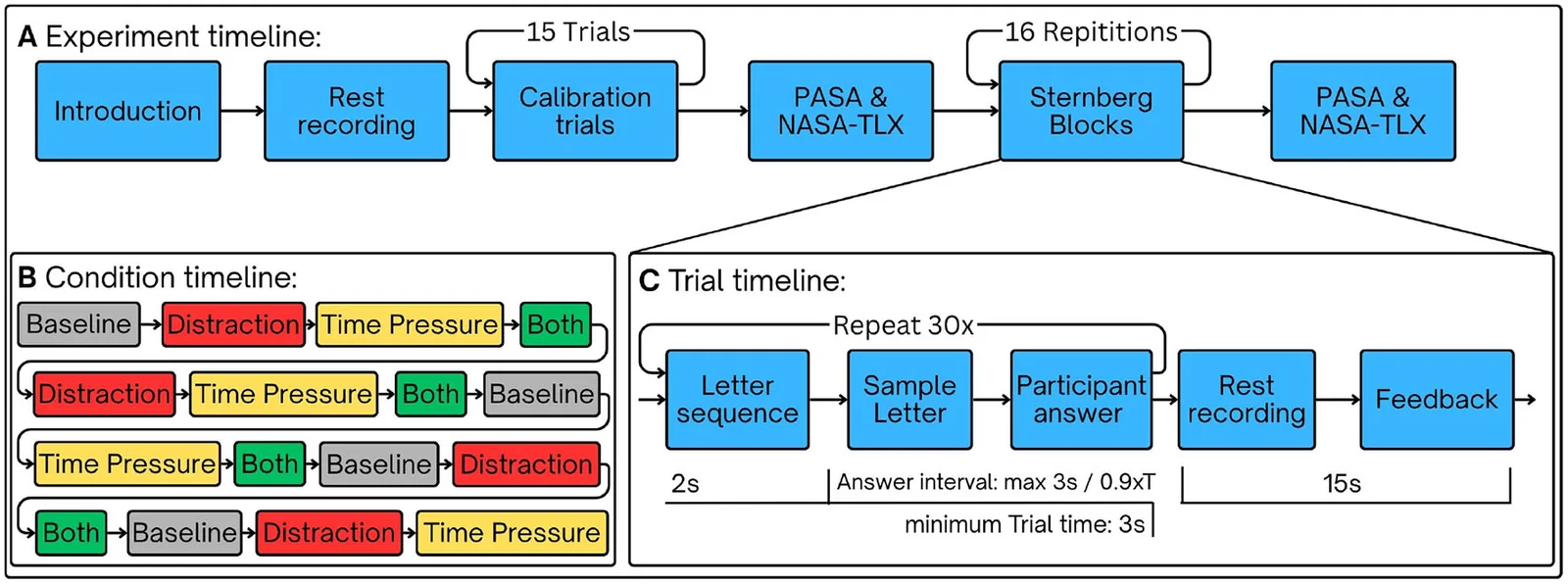
Experimental procedure of the study. **(A)** Overall experimental timeline including resting state recordings calibration, questionnaires, and task blocks. **(B)** Counterbalanced order of the experimental conditions: “Baseline” (Gray), “Auditory distraction” (red), “Time pressure” (yellow) and simultaneous time pressure and auditory distraction “Both” (green) **(C)** Structure of a single Sternberg task block and trial. The sample letter sequence was displayed for 2 s, followed by one target letter which was visible for the period available for answering. This period was defined by either 90% of the averaged answer period during the calibration trials (Time pressure condition) or a maximum of 3 s. In any case, the screen remained blank until a total trial time of 3 s.

The working memory paradigm was based on the Sternberg task (Sternberg, 1969). The experiment was based on a modified Sternberg working memory task, in which participants encoded a set of letters, maintained them over a brief retention interval, and subsequently indicated whether a probe letter had been part of the memorized set. Task difficulty was standardized across conditions such that each trial consisted of a memory set containing four to six letters, followed by a single probe letter requiring a recognition response if the single letter was contained in the memory set. Prior to the experimental conditions, an individual calibration trial block was performed to estimate each participant's baseline response time. These response times were subsequently used to define participant-specific response deadlines during the time-pressure conditions, ensuring that the manipulation imposed a comparable level of temporal demand across individuals despite inter-individual differences in baseline processing speed. Throughout all conditions, participants were asked to answer conscientious correct while maintaining a quick response speed. In the baseline condition, the letter sequence was presented for 2 s, after which the probe letter appeared for a maximum duration of 3 s or until a response was given. The *time-pressure* condition was designed to induce cognitive demand by reducing the available response interval to 90% of the participant-specific average response time obtained during calibration. The *distraction* condition presented prerecorded environmental sounds from office and café environments through headphones while maintaining the response timing of the baseline condition. Care was taken to position the headphones without affecting the EEG montage. The auditory distraction consisted of prerecorded office and caf ambience comprising ecologically valid everyday sounds such as background conversations, cutlery noise, telephone ringing, keyboard and mouse clicks, footsteps, and other typical workplace activities. They were not explicitly validated for distractibility. The *combined* condition simultaneously included both time pressure and auditory distraction. To ensure comparable trial lengths, each trial was followed by a blank-screen interval such that the total trial duration was at least 3 s. During the time-pressure and combined conditions, responses were only accepted within the designated response window and were not possible during the subsequent blank-screen interval.

To further increase perceived task demands, participants received performance feedback after each block and resting-state recording. The displayed score was systematically reduced by 15% relative to the actual performance. Participants were informed about this manipulation during the final debriefing after completion of the experiment.

### 2.2 Participants

Data were acquired from 30 healthy participants (15 female, 15 male; age: 24.83 ± 3.45 years) who provided written informed consent prior to participation. All participants reported normal or corrected-to-normal vision. During the experiment, participants were instructed to remain seated and to minimize eye, head, and body movements to reduce artifacts. The study was approved by the local ethics committee of Graz University of Technology (GZ: EK-114/2026) and conducted in accordance with the Declaration of Helsinki.

### 2.3 Questionnaires

Subjective stress and mental workload were assessed using the Primary Appraisal Secondary Appraisal questionnaire (PASA by Gaab, 2009) and a slightly adapted version (Scale from 1-10) of the NASA Task Load Index (NASA-TLX by Hart and Staveland, 1988), respectively. The PASA is based on the transactional stress model proposed by (Lazarus and Folkman 1985). It evaluates the perceived relationship between situational demands and available coping resources by assessing both primary appraisal (perceived challenge and threat) and secondary appraisal (perceived control and coping capabilities). The resulting appraisal reflects the balance between perceived task demands and available coping strategies. The NASA-TLX is a widely established instrument for assessing subjective mental workload during task performance. Higher NASA-TLX scores indicate greater perceived workload, higher PASA scores represented higher perceived stress. Both questionnaires were presented directly on screen and completed electronically. Responses were provided using Likert scales ranging from 1–6 for the PASA and 1–10 for the NASA-TLX. Questionnaire scores obtained before and after the experiment were compared using paired-samples t-tests. Behavioral performance was analyzed using mixed-effects models. Response times were analyzed with a linear mixed-effects model, whereas response accuracy was analyzed using a binomial logistic mixed-effects model. In both models, experimental condition and within-condition repetition were included as fixed effects, and participant was included as a random effect.

### 2.4 Data recording

EEG data were recorded using a LiveAmp amplifier (Brain Products GmbH, Gilching, Germany) with 32 active electrodes sampled at 500 Hz. Electrode positions followed the international 10–10 system and are illustrated in Figure 2. The ground electrode was placed at the left mastoid and the reference electrode at the right mastoid. During EEG preparation, electrode impedances were maintained below 20 kΩ. Prior to the start of the experiment, the incoming EEG signal was visually inspected to ensure adequate signal quality and stable electrode contacts. The experimental task was presented on a 24-inch monitor positioned approximately 1 m in front of the seated participant. Stimulus presentation and response acquisition were implemented using PsychoPy (v2025.1.3). Experimental markers, representing the stimuli onsets and timeline of the experiment were synchronized with the EEG recordings using the Lab Streaming Layer (LSL) framework (Kothe et al., 2025).

**Figure 2:**
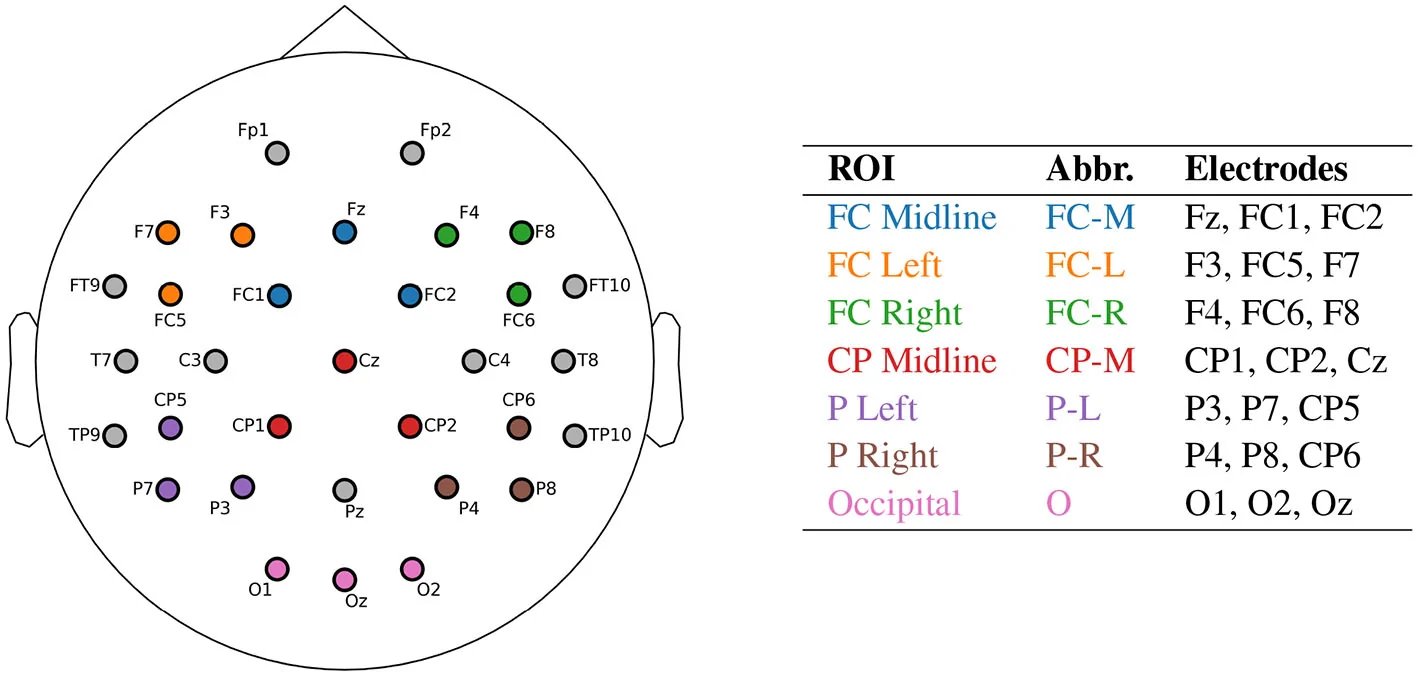
EEG sensor layout according to the international 10–10 system. Colored regions indicate the regions of interest (ROIs) used for connectivity analysis. The corresponding electrode assignments are listed on the right.

For functional connectivity analysis, individual electrodes were aggregated into predefined regions of interest (ROIs) as listed in Figure 2. ROI-based aggregation reduces channel-level noise, improves interpretability, and decreases the dimensionality of the connectivity analysis. The selected ROIs were designed to represent broad anterior, posterior, lateralized, and occipital scalp regions while preserving hemispheric organization. As frontoparietal interactions are particularly relevant for working memory, attentional allocation, and cognitive control, the ROI definition was chosen to maintain a separation between frontal and posterior regions. Furthermore, all ROIs contained an equal number of electrodes to ensure comparable spatial averaging.

### 2.5 Preprocessing

All EEG data were processed using Python 3.12, primarily relying on the MNE-Python toolbox (Gramfort et al., 2013). Additionally, we used the following packages: mne-connectivity (0.8), numpy (2.4.4), pandas (2.2.3), scipy (1.17.1, and statsmodels (0.14.6). First, event markers were aligned to the temporally closest EEG sample to minimize timing jitter. The continuous recordings were then band-pass filtered between 1 and 75 Hz using a fourth-order Butterworth filter. Subsequently, Independent Component Analysis (ICA; 15 components, extended Infomax) was applied to identify and remove ocular and muscular artifacts. The ICA decomposition was restricted to 15 components because these components accounted for more than 95% of the explained variance across recordings. This dimensionality was therefore considered sufficient to retain the dominant signal structure required for identifying ocular and muscular artifacts while avoiding decomposition into additional low-variance components. EOG-related components were identified manually based on characteristic blink and eye-movement patterns, typically exhibiting frontal topographies. EMG-related components were detected using MNE's automated muscle artifact detection, which evaluates three criteria: (i) a positive log-log spectral slope between 7 and 45 Hz, (ii) increased peripheral component power, and (iii) low spatial smoothness indicating focal activity. This procedure led to an average of 6.3 removed components throughout all datasets.

After artifact removal, a 50 Hz notch filter was applied to suppress power-line interference. Recordings were initially acquired using a mastoid reference and subsequently re-referenced to the common average reference. The cleaned signals were then band-pass filtered between 1 and 40 Hz using a fourth-order Butterworth filter. Finally, the data were segmented into 3 s epochs relative to memory-set onset. A 3 s epoch length was selected to capture the complete memory-set encoding period and the early maintenance phase while providing sufficient temporal resolution for reliable spectral and functional connectivity estimation, particularly in the lower frequency bands. Furthermore, a fixed epoch duration ensured comparable analyses across all experimental conditions. Epochs were rejected if the signal was flat (< 1μV) or exceeded a peak-to-peak amplitude of 130μV which resulted into an average of 2.5 rejected epochs per participant. The complete preprocessing pipeline is illustrated in Figure 3.

**Figure 3:**
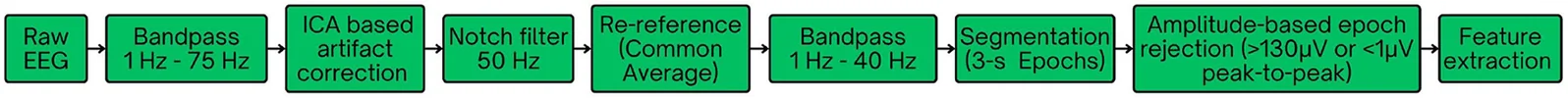
Overview of the EEG preprocessing pipeline: The raw EEG was band-pass-filtered (1 Hz–75 Hz), followed by an ICA-based artifact correction, notch filtering, re-referencing to the common average, band-pass-filtering (1 Hz–40 Hz), segmentation into 3-s epochs, amplitude-based epoch rejection, and subsequent feature extraction.

### 2.6 Feature extraction and statistical analysis

Bandpower (BP) features were extracted from each epoch using power spectral density (PSD) estimation based on Welch's method (500 samples per segment, 50% overlap, Hann window, 1,024-point FFT). Spectral power was computed between 1 and 40 Hz. Subsequently, average bandpower values were extracted for the delta (1–3.5 Hz), theta (4–7.5 Hz), alpha (8–12.5 Hz), and beta (13–20 Hz) frequency bands at each electrode.

Hemispheric asymmetry was quantified as the difference between logarithmically transformed (log_10_) bandpower values of homologous electrode pairs. Frontal alpha asymmetry (FAA) and frontal beta asymmetry (FBA) were calculated for the electrode pairs Fp1–Fp2, F7–F8, F3–F4, FT9–FT10, FC5–FC6, C3–C4, TP9–TP10, CP5–CP6, CP1–CP2, P7–P8, P3–P4, and O1–O2.

To identify condition-specific neural modulations, we employed topographical t-maps as previously described by (Wriessnegger et al. 2024, 2025). Linear mixed-effects (LME) models (Pinheiro and Bates, 2000) were fitted separately for each electrode and frequency band using the MixedLM implementation from the statsmodels package. The z-scored bandpower values served as dependent variables, whereas experimental condition was included as a fixed effect and participant as a random effect. The same procedure was applied to the asymmetry measures after z-score normalization. Resulting p-values were corrected for multiple comparisons across electrodes using the Benjamini–Hochberg False Discovery Rate (FDR) procedure (Benjamini and Hochberg, 1995).

For visualization, the t-statistics obtained from the LME models were projected onto the scalp to generate topographical statistical maps (t-maps). These maps provide a spatial representation of condition-related neural modulations and facilitate the interpretation of statistically significant effects. In addition to contrasts between each experimental condition and the baseline condition, pairwise contrasts between the experimental conditions were extracted from the same LME framework and visualized as t-maps To improve interpretability and avoid unnecessarily complex interaction terms, separate models were fitted for each dependent variable.

Condition-dependent changes in functional connectivity were assessed using the weighted Phase Lag Index (wPLI). Band-specific EEG signals served as input for connectivity estimation. The wPLI was selected because it reduces sensitivity to spurious zero-lag synchronization caused by volume conduction and common reference effects (Vinck et al., 2011). Connectivity estimates were obtained using multitaper spectral estimation and subsequently aggregated at the ROI level. To emphasize condition-related effects, connectivity values were expressed as percentage changes relative to the baseline condition according to Equation 1:


$$\begin{array}{l} \delta \text{wPLI}\left(\%\right)=\frac{\text{wPL}{\text{I}}_{\text{condition}}-\text{wPL}{\text{I}}_{\text{baseline}}}{\text{wPL}{\text{I}}_{\text{baseline}}}\times 100. \end{array}$$ (1)

where C denotes the ROI-to-ROI wPLI value.

Statistical analyses of functional connectivity were performed using LMEs fitted separately for each of the 21 unique ROI-to-ROI connections derived from the seven predefined regions of interest. The epoch-wise weighted phase lag index (wPLI) served as the dependent variable, allowing the repeated-measures structure of the data to be retained. Condition was included as a fixed effect, while participant was modeled as a random effect to account for within-subject dependence. To control for multiple testing across the 21 ROI connections, the resulting p-values were adjusted using the FDR procedure. Statistical significance was defined as an FDR-adjusted *q*-value of < 0.05. Connectivity matrices displaying percentage changes relative to baseline are presented for descriptive visualization only and were not used for statistical inference.

## 3 Results

### 3.1 Behavioral analysis

Figure 4 displays the behavioral performance and subjective ratings obtained from the PASA and NASA-TLX questionnaires. The upper left panel shows the response times (RT) across experimental runs. Response times increased slightly during the second run before stabilizing at approximately 0.6–0.7 s for the remainder of the experiment. The upper right panel displays the average accuracies. Overall accuracy remained high across all runs, generally exceeding 95% correctness. Periodic decreases in performance were observed during several runs, with accuracies dropping to approximately 85%. The lower panels show the average PASA and NASA-TLX scores before and after the experiment. PASA scores exhibited a small increase from pre- to post-experiment measurements (*p*_*fdr*_ = 0.09, t = –1.765, 95% CI = [–0.08479, 1.151]). In contrast, NASA-TLX scores increased *p*_*fdr*_ ≤ 0.001, t = –3.755, 95% CI = [0.2527, 0.8573] over the course of the experiment.

**Figure 4:**
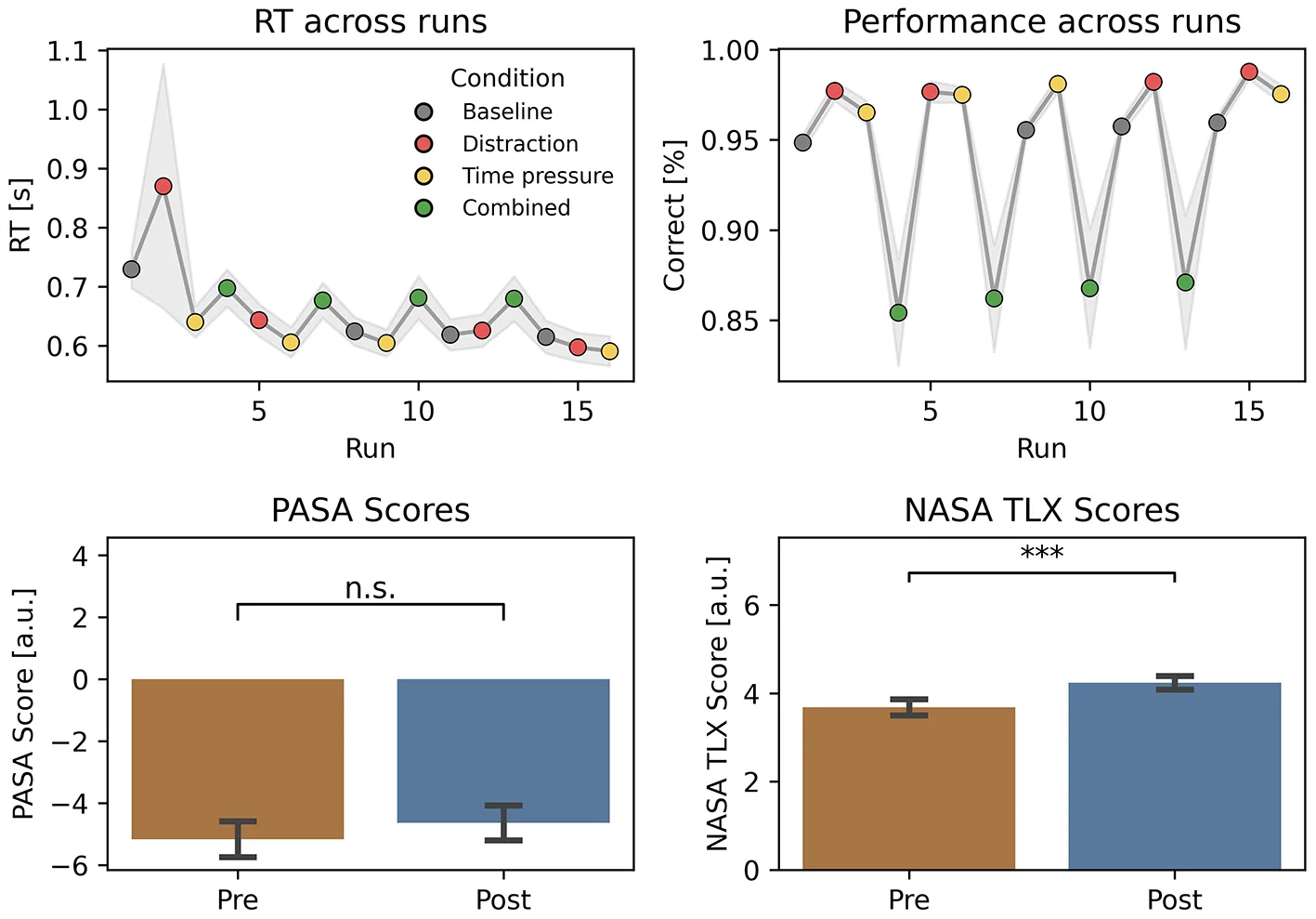
Overview of behavioral performance and subjective ratings. The upper left panel shows average response times (RT) across runs, whereas the upper right panel displays average accuracies. The lower panels show PASA and NASA-TLX scores before and after the experiment. Shaded areas (RT and performance) represented standard deviation, error bars (PASA and NASA-TLX) represent the standard error. Significant changes are marked with asterisks.

Table 1 summarizes the mixed-effects model results for response time and accuracy. Response time showed a significant decrease across block repetitions (β = −0.035, *p* = 0.001), indicating a practice effect, whereas no significant effects of experimental condition were observed. For accuracy, distraction (β = 0.939, *p* < 0.001), and time pressure (β = 0.610, *p* < 0.001) were associated with increased accuracy relative to baseline, whereas the combined condition showed significantly reduced accuracy (β = −1.277, *p* < 0.001).

**Table 1:** Mixed-effects models of reaction time and accuracy.

Outcome	Predictor	Estimate β	95% CI	*p*
Reaction time	Distraction	0.037	[−0.029, 0.104]	0.272
	Time pressure	−0.036	[−0.103, 0.030]	0.285
	Combined	0.037	[−0.030, 0.104]	0.277
	Repetition	−0.035	[−0.056, −0.014]	0.001
Accuracy	Distraction	0.939	[0.694, 1.184]	< 0.001
	Time pressure	0.610	[0.399, 0.821]	< 0.001
	Combined	−1.277	[−1.375, −1.178]	< 0.001
	Repetition	0.087	[0.059, 0.115]	< 0.001

Baseline was the reference condition. All estimates are fixed-effect coefficients (β). For the reaction-time model, coefficients are expressed in seconds. For the accuracy model, coefficients are expressed on the log-odds scale. Both models included a participant-specific random intercept.

### 3.2 Bandpower changes

Figure 5 shows the average bandpower (BP) across all standard frequency bands. In Figure 5a, the first row displays the absolute power distribution (dB), whereas the modified conditions are shown as relative percentage changes with respect to the baseline condition. Right below, Figure 5b represents the ROI-specific oscillatory change (see Figure 2, right column, for definition) aggregated BP changes. The topomaps illustrate the spatial distribution of condition-dependent effects, whereas the ROI summaries quantify the average bandpower changes within the predefined ROIs. The maps are masked with black markers indicating statistically significant effects (α < 0.05) after correction for multiple comparisons using FDR.

**Figure 5:**
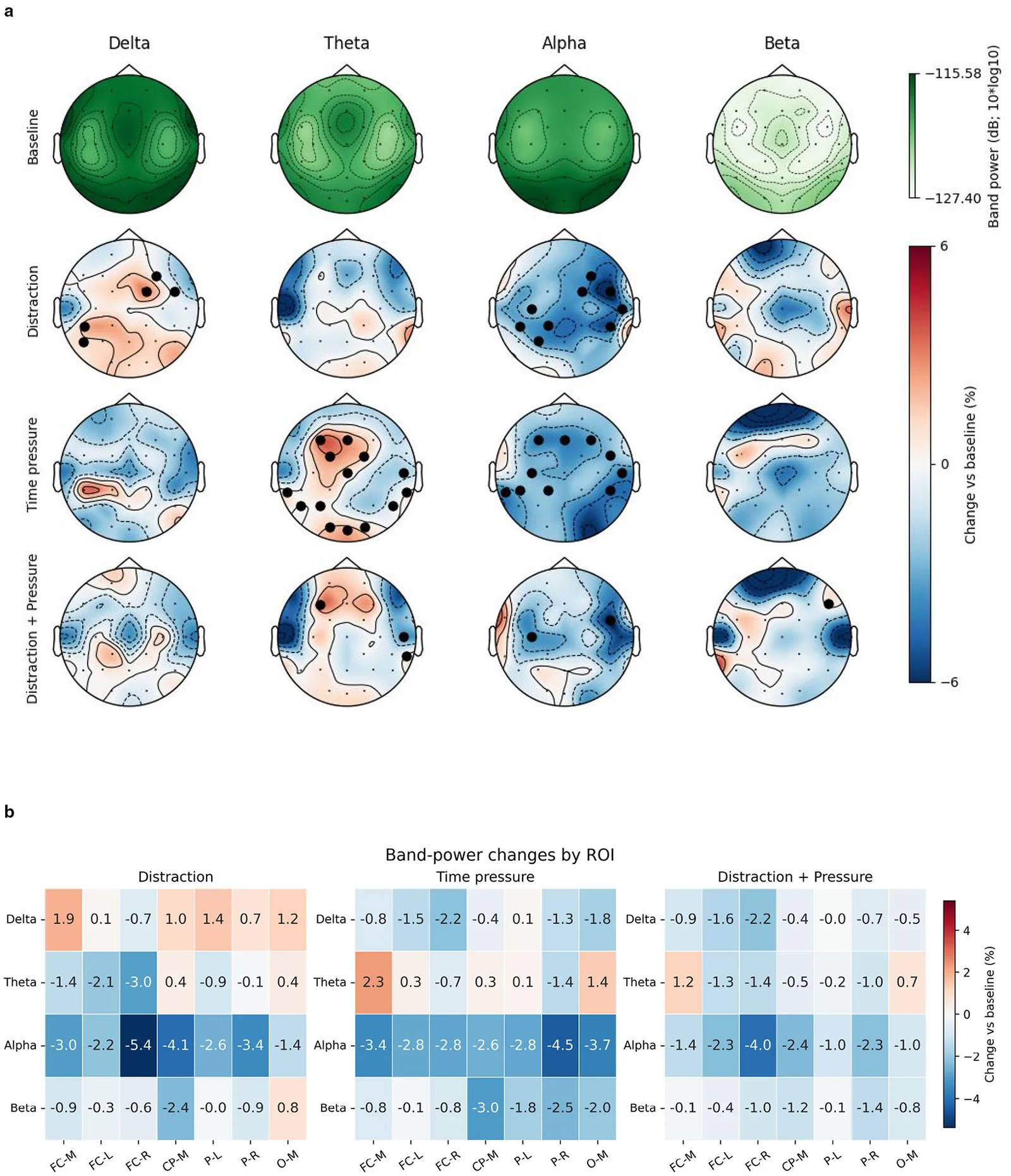
Topographic maps illustrate percentage bandpower changes relative to baseline and are displayed after significance masking based on the statistical analyses. The lower panel summarizes ROI-averaged bandpower changes for descriptive visualization; no separate statistical testing was performed on these aggregated values. **(a)** Bandpower topomaps for all experimental conditions and frequency bands. The first row shows the absolute baseline bandpower distribution (dB), whereas the remaining rows depict relative bandpower changes (%) with respect to the baseline condition. All electrode positions marked with a black dot indicate significant effects after correction for multiple comparisons (α ≤ 0.05). **(b)** Summary of the ROI-averaged bandpower changes (% vs. baseline) for visualization purposes.

In the baseline condition, the delta band exhibits central, lateral, and occipital activity. Theta shows a predominantly frontocentral midline distribution, while alpha exhibits its strongest activity in parietal and occipital regions. Beta also shows pronounced oscillatory power in frontocentral midline regions as well as in occipital areas.

In the distraction condition, significant delta bandpower increases are observed around F4, FC2, and FC4 as well as in P7 and CP5 (FC–M: +1.9%; P–L: +1.4%). Significant alpha power decreases were found in lateral right electrode positions (FC–R –5.4%) and left centro-parietal electrodes (P–L –2.6%). A significant alpha-band decrease was also observed at CP6 (P–R –3.4%). No statistically significant theta-band effects were observed, although lateral decreases in frontal regions were apparent. The beta band shows non-significant power decreases in left frontal and central regions.

The time-pressure condition induced significant theta-bandpower increases in frontal midline regions spanning from F3 to FC2 (FC–M: +2.3%). Additional significant, but less pronounced, increases were observed in occipital electrodes (O–M: +1.4%) together with minor changes in lateral parietal regions (P–R: –1.4%). No statistically significant delta-band changes were observed, although moderate decreases were apparent in right frontocentral and occipital regions. Likewise, no statistically significant beta-band changes were identified despite localized decreases around Fp1/Fp2 and the centro-parietal midline

In the combined condition, we found few significant changes: the theta band indicated a significant increase in F3 (FC–M: +1.2%) as well as significant decreases in T8 and TP10 (P-R: –2.3%). Significant alpha band decreases were observed in C3 (FC–L: –2.3%) and FC6 (FC–R: –4.0%). A small but statistically significant beta-band increase was observed at F8 (ROI summary: +1.0%). Non-significant effects included left temporal BP theta decreases, a global decrease in the alpha band and a pronounced beta decrease in Fp1 and Fp2.

### 3.3 t-maps

Figure 6 displays the topographical t-maps as described in the methods section.

**Figure 6:**
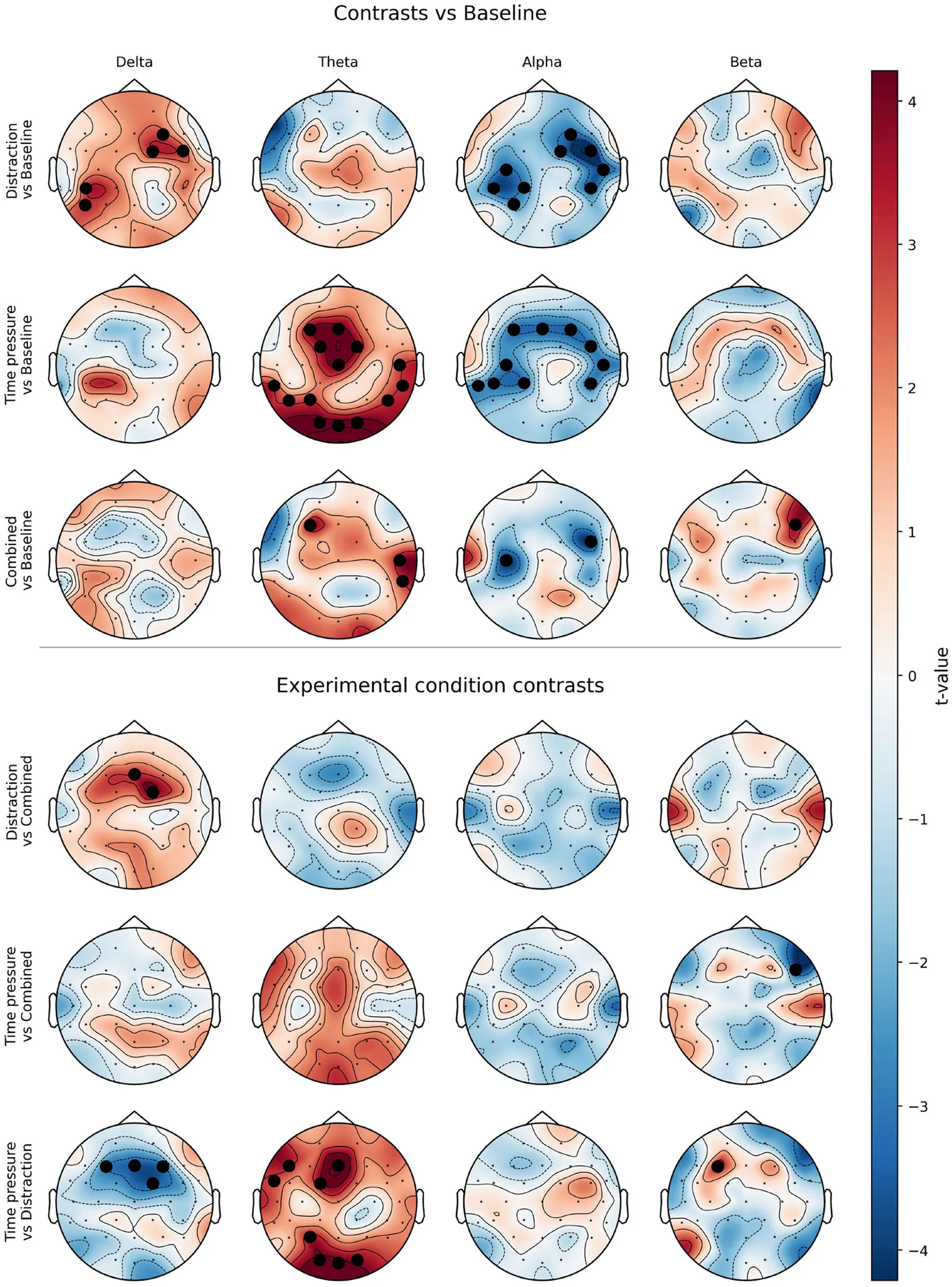
Topographical t-maps showing condition-related effects across frequency bands. Black markers indicate electrodes with statistically significant effects after FDR correction.(α < 0.05). “Distraction” represents the auditory distraction condition, “Time pressure” the condition under time pressure and “Combined” the concurrent presentation of both stimuli.

#### 3.3.1 Contrast vs. baseline condition

The distraction condition exhibits moderate positive *t*-values in the delta band, reaching (*t* ≈ 3) over the right frontal and left temporoparietal regions, where statistically significant effects are observed. Likewise, the alpha band is characterized by dominant negative *t*-values (*t* ≈ –4) forming bilateral lateralized clusters extending from central to parietal electrode rows. Theta and beta bands exhibit comparatively weak and spatially diffuse *t*-value distributions without statistically significant effects.

Time pressure induces the overall most dominant positive *t*-values in the theta band, with significant electrode positions located in frontal to central midline regions as well as occipital and symmetric parietotemporal areas. In the alpha band, the frontal electrode row from F3 to F4 shows significant negative t-values, in addition to significant effects in left- and right-hemispheric lateralized electrodes within the central and centroparietal rows. Descriptively, the beta band exhibits an inverse spatial pattern, although no effects reach statistical significance.

The combined condition of time pressure and distraction shows positive t-values in the theta band within frontal to central midline regions, including a single significant electrode, as well as significant effects with higher *t*-values (*t* ≈ 3.5) in right temporal regions. Similarly, the alpha band shows two spatially isolated significant electrodes (*t* ≈ –3.5). The beta band also contains only a single significant electrode, while exhibiting a ring-shaped pattern of positive *t*-values around Cz that does not reach statistical significance.

#### 3.3.2 Experimental condition contrasts

Relative to the combined condition, distraction is characterized by moderate positive delta-band *t*-values (*t* ≈ 3) over frontal regions together with a localized cluster of statistically significant electrodes (Fz, F2). Theta, alpha, and beta bands display comparatively weak and spatially restricted *t*-value differences. Relative to the combined condition, the time-pressure condition exhibits a single significant electrode FC8 showing a pronounced decrease (*t* ≈ –4). In addition, non-significant moderately strong positive theta-band *t*-values (*t* ≈ 2.5–3.5) are noted. Differences in the remaining frequency bands are characterized by comparatively weak and spatially localized *t*-value distributions. Direct comparisons between the experimental conditions showed significant frontal delta decreases in F3, Fz, F4 and FC2 (*t* ≈ -3) while theta significantly increased in Fz and FC1 (*t* ≈ +4). Additional significant theta increases were found in the occipital row (*t* ≈ +4) and in F7 & FT9 (*t* ≈ +3.5). The beta band shows a significant effect in F4 (*t* ≈ +3) whereas the alpha map did not show significant effects.

### 3.4 Hemispheric asymmetry

The asymmetry metrics are visualized in Figure 7. The maps are masked with black markers indicating statistically significant effects (α < 0.05) after correction for multiple comparisons using FDR.

**Figure 7:**
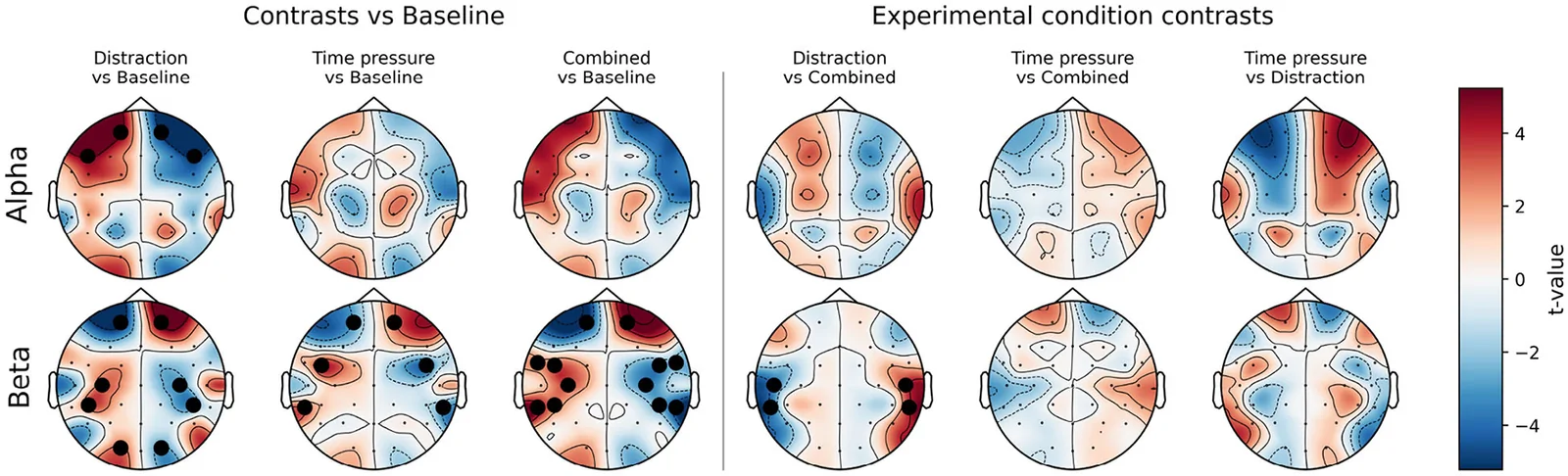
t-maps of hemispheric alpha and beta asymmetry across experimental conditions. Colors represent t-values obtained from the linear mixed-effects models. Black markers indicate electrodes with statistically significant effects after FDR correction (α < 0.05). “Distraction” represents the auditory distraction condition, “Time pressure” the condition under time pressure and “Combined” the concurrent presentation of both stimuli.

Relative to baseline, hemispheric alpha asymmetry was characterized by predominantly negative *t*-values in the right frontal hemisphere, reaching statistical significance only for the F7–F8 and Fp1–Fp2 electrode pairs during the distraction condition. The “Combined vs. Baseline” contrast showed a non-significant decrease in the right hemisphere (*t* ≈ -3.5) and “Time pressure vs. Distraction” a frontal increase (*t* ≈ +4). In contrast, hemispheric beta asymmetry revealed significant asymmetry effects across all baseline contrasts and the “Distraction vs. combined” contrast. Significant effects were observed for the Fp1–Fp2 pair in all baseline contrasts (positive *t*-values ranging from *t* = 2.5–4). Furthermore, comparing the “Distraction vs. Baseline” contrast, we found decreased *t*-values (*t* ≈ –3) in the C3–C4 and CP5–CP6 electrode pairs and spatially separated, in the O1–O2 pair. The contrast “Time pressure vs. Baseline” displayed significant effects in the pairs FC5–FC6 and TP9–TP10 with negative *t*-values (*t* ≈ –3). During the “Combined vs. baseline” contrast, we found additional ring-shaped clusters of significant electrodes around (and not including) the T7–T8 pair with negative t-values (*t* ≈ –3). The experimental condition contrasts show significant effects in the contrast map “Distraction vs. combined.” Temporal pairs (T7–T8 and TP9–TP10) show t-value increases in right hemisphere positions (*t* ≈ +4).

### 3.5 Functional connectivity

Figure 8 shows the descriptive functional connectivity between the predefined ROIs, expressed as percentage changes relative to baseline. The connectivity matrices are presented for descriptive visualization only to illustrate the spatial distribution of the observed changes. Statistical inference based on the linear mixed-effects analyses did not identify any statistically significant condition effects for any ROI-to-ROI connection after false discovery rate correction (all *q*>0.05).

**Figure 8:**
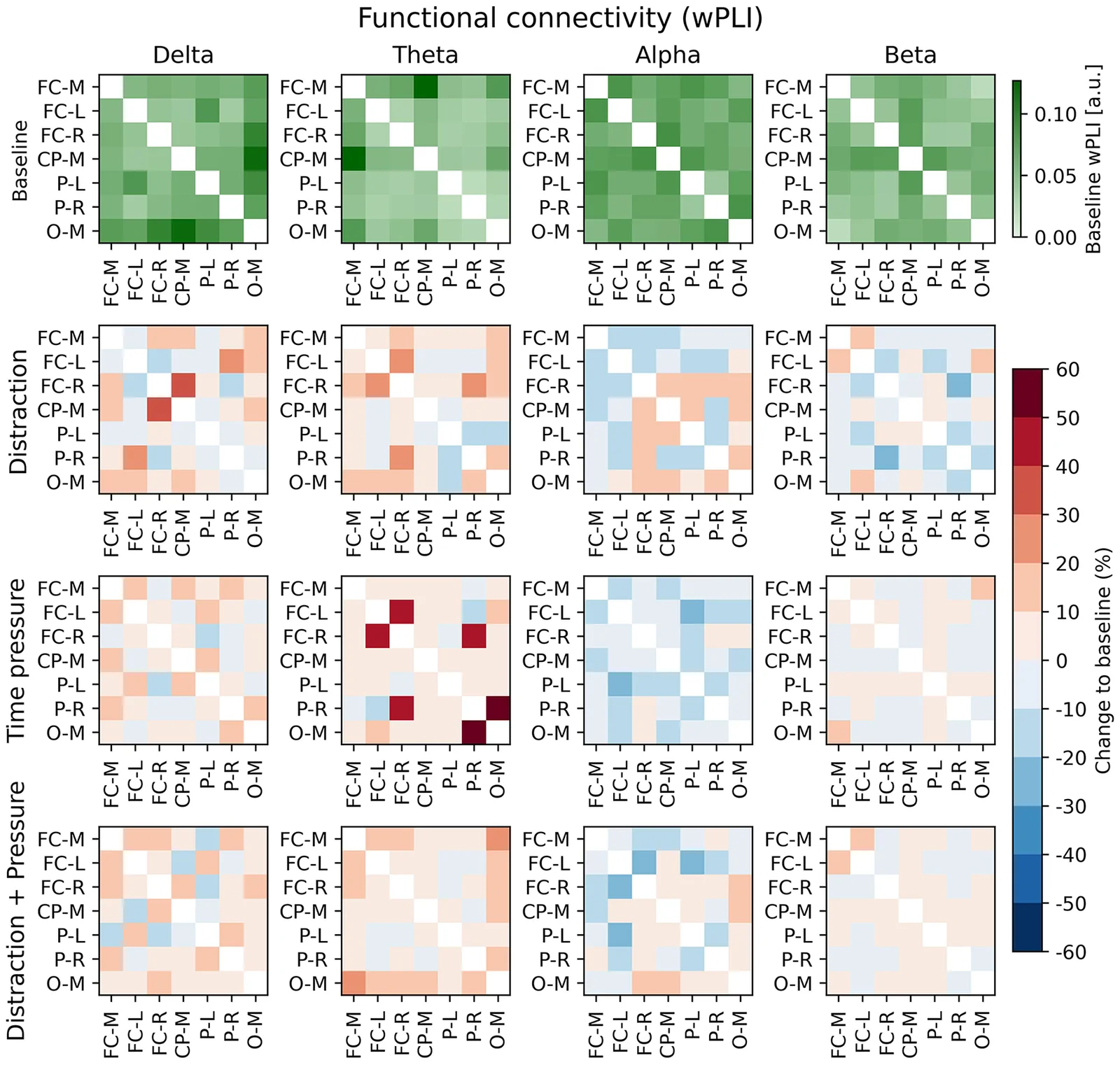
wPLI-based functional connectivity between the predefined ROIs across frequency bands and experimental conditions. Baseline connectivity is shown in the first column, while the remaining matrices represent percentage changes relative to baseline. The connectivity matrices are presented for descriptive visualization of the observed connectivity patterns. Statistical inference was performed using linear mixed-effects models and is reported separately in the text.

In the delta band, the baseline condition is characterized by stronger coupling between CP–M, P–R/P–L, and O–M regions, as well as between FC-L and P-L regions and between FC-R and O–M ROIs. Relative to baseline, the descriptive connectivity maps suggest regional changes involving fontocentral, fronto-parietal, and right-hemispheric connections. The theta band is dominated by strong baseline coupling between the FC–M and CP–M regions. Descriptively, the experimental conditions are characterized by increased frontocentral and right-hemispheric connectivity together with reduced P–L/O–M coupling. The alpha band exhibits a comparatively homogeneous baseline connectivity pattern. Relative to baseline, the descriptive maps indicate moderate regional changes across conditions, predominantly involving reduced left-hemispheric and frontal connectivity together with isolated increases in FC-R/O-M connections. In the beta band, baseline connectivity is primarily centered on the CP-M region. Descriptive changes across conditions are comparatively small and mainly involve FC–M, and FC–R to P–R connections. Statistical inference based on the linear mixed-effects analyses did not identify any statistically significant condition effects for any of the 21 ROI-to-ROI connections after false discovery rate correction (all *q*>0.05). Accordingly, the observed connectivity patterns are not interpreted as evidence of reliable condition-dependent functional connectivity changes.

## 4 Discussion

### 4.1 Behavioral analysis

Behavioral performance differed across conditions primarily in terms of accuracy rather than response time. Mixed-effects modeling showed that response times were unaffected by experimental condition but decreased significantly across repetitions, consistent with a practice effect. Accuracy increased during distraction and time pressure relative to baseline but decreased in the combined condition, indicating that performance was maintained or improved under the individual manipulations but deteriorated when auditory distraction and time pressure occurred simultaneously. Subjective workload increased significantly according to NASA-TLX scores, whereas PASA scores showed only a non-significant tendency toward higher values. Together, these findings suggest that participants generally maintained response speed under increased cognitive demands, but the combined condition exceeded this compensation and resulted in reduced accuracy.

### 4.2 Bandpower changes

The alpha band was hypothesized to be primarily associated with attentional allocation, information filtering, and distractor suppression. Accordingly, a widespread reduction in alpha power was expected under distraction. The observed results were consistent with this hypothesis, showing a global decrease in alpha bandpower with a stronger effect in the right hemisphere. In particular, right-hemispheric alpha suppression and left lateral centroparietal bandpower decreases reached significance. In the experimental contrasts, distraction was further characterized by distinct frontal delta activity relative to the combined condition, whereas alpha-band differences between the experimental conditions remained comparatively weak. These findings are consistent with alpha-mediated attentional filtering and the maintenance of task-relevant information (Zanto and Gazzaley, 2009; Bonnefond and Jensen, 2012; Gutteling et al., 2022).

Additional observations included moderate delta-bandpower increases in right frontal and left parietal regions, which reached significance during distraction. In the experimental contrasts, frontal delta activity further differentiated distraction from the combined condition. These effects may reflect resource allocation processes (Harmony, 2013; López et al., 2019) and working memory maintenance (Tóth et al., 2012; Baddeley, 2003).

No significant theta effects were observed, although moderate frontal decreases were apparent descriptively. The absence of changes in theta is reasonable, as auditory distraction primarily affects attentional filtering rather than pronounced recruitment of executive control networks (Cavanagh and Frank, 2014).

The beta band showed decreases in left frontal and central regions without reaching significance. Likewise, the experimental contrasts revealed only weak beta-band differences between distraction and the remaining conditions. Nevertheless, these tendencies may indicate altered task engagement and increased processing demands, although such interpretations should be treated with caution given the limited statistical support (Engel and Fries, 2010; Spitzer and Haegens, 2017). Overall, the alpha-band findings are consistent with the proposed role of attentional filtering and distractor suppression during auditory distraction.

We further hypothesized increased frontal theta activity under time pressure, reflecting enhanced cognitive control and executive coordination. In the time-pressure condition, theta showed the largest observed bandpower and wPLI changes between conditions. Significant frontal-midline and occipital bandpower increases were observed. Furthermore, the experimental contrasts revealed significant frontal theta increases relative to distraction and descriptively stronger, but non-significant, effects relative to the combined condition. The observed frontal-midline theta increase is consistent with previous reports associating frontal-midline theta with increased cognitive control demands. Frontal-midline theta has repeatedly been associated with executive control and attentional regulation (Cavanagh and Frank, 2014). More recently, (Yan et al. 2024) reported increased frontal and frontocentral theta activity during acute stress exposure, suggesting compensatory recruitment of control processes to maintain performance. This association cannot be confirmed in the present work due to the absence of significant PASA changes, although a descriptive increase was observed. The simultaneous frontal and parietal activation observed here may therefore reflect both working memory maintenance and neural responses to cognitive demand (Muthukrishnan et al., 2020). Especially in the time-pressure condition, parietal theta increased more strongly than during distraction, which might be associated with increased working memory resource recruitment (Chang et al., 2023).

The global decrease in alpha bandpower reached significant positions in the frontocentral electrode row as well as in lateral centroparietal electrodes. In the experimental contrasts, alpha-band differences between time pressure and the remaining experimental conditions were comparatively small despite the widespread suppression observed relative to baseline. Consistent with this spatial distribution, the asymmetry analysis revealed stronger right-frontal alpha suppression than during distraction. Rather than reflecting selective sensory filtering, this pattern may indicate increased cortical engagement and sustained attentional allocation under cognitive demand (Klimesch, 1999; Gutteling et al., 2022). Similar widespread oscillatory changes under increased stress-related demands have also been reported by (Yan et al. 2024).

The beta band showed a decrease in bandpower around Fp1, whereas the adjacent frontocentral electrode row exhibited small increases. Experimental contrasts likewise revealed only isolated beta-band differences between conditions. Although not significant, these frontocentral beta tendencies are noteworthy, as beta oscillations have been linked to top-down attentional regulation and cognitive adaptation under increased task demands (Palacios-García et al., 2021; Engel and Fries, 2010). The spatial relationship between alpha and beta activity appears as opposite oscillatory changes and may therefore reflect complementary mechanisms supporting performance under time pressure.

The combined condition did not simply amplify isolated oscillatory effects but instead exhibited a distinct spatial pattern of oscillatory changes. Theta activity remained elevated in frontal regions and showed significant decreases in right temporal electrodes, whereas alpha suppression persisted with only isolated significant electrodes. A small frontal beta increase represented the only significant beta-band effect. In the experimental contrasts, the combined condition differed from distraction by reduced frontal delta activity and from time pressure by weaker frontal theta recruitment, indicating that the simultaneous occurrence of both manipulations resulted in a distinct oscillatory organization rather than an additive response.

Diffuse delta-band changes might suggest large-scale coordination, even if not significant. Elevated frontal theta-band power may indicate continued recruitment of cognitive control processes (Yan et al., 2024; Muthukrishnan et al., 2020). Alpha suppression persisted and was only significantly lateralized than during distraction, suggesting that concurrent auditory distraction and time pressure altered the spatial organization of attentional processes rather than simply strengthening alpha suppression (Gutteling et al., 2022; Zanto and Gazzaley, 2009). Beta-related changes remained exploratory and may reflect interactions between stress-related executive processing and simultaneous auditory stimulation (Spitzer and Haegens, 2017; Palacios-García et al., 2021).

### 4.3 Hemispheric asymmetry

Our hypothesis included enhanced right-hemispheric alpha suppression and diffuse asymmetric patterns in the beta band. Alpha asymmetry reached statistical significance only during the distraction condition, where frontal electrode pairs exhibited pronounced right-hemispheric asymmetry. Although progressively increasing *t*-values were observed during the time-pressure and combined conditions, these contrasts did not reach statistical significance. Furthermore, the experimental contrasts revealed opposing frontal *t*-value patterns between distraction and time pressure, indicating distinct lateralization profiles despite comparable alpha-band suppression in the bandpower maps.

These findings suggest that hemispheric alpha asymmetry was primarily associated with auditory distraction rather than increased cognitive demand alone. This interpretation is consistent with previous reports relating frontal alpha modulation to attentional filtering, sensory filtering, and distraction prediction (Bonnefond and Jensen, 2012; Wriessnegger et al., 2024). While alpha suppression was observed across all conditions, significant lateralization remained largely restricted to distraction, suggesting that widespread alpha-band changes do not necessarily coincide with pronounced hemispheric asymmetry.

Beta asymmetry exhibited a more spatially distributed organization and, in contrast to alpha, reached statistical significance across all baseline contrasts. Significant frontal asymmetry was consistently observed, while additional condition-dependent effects extended toward central, centroparietal, temporal, and occipital electrode pairs. The experimental contrast between distraction and the combined condition further revealed significant temporal asymmetry differences.

In contrast to the largely non-significant beta-bandpower maps, the consistent beta asymmetry effects may suggest that hemispheric differences in beta activity were more sensitive to the experimental manipulations than regional beta-bandpower alone. This interpretation is compatible with the proposed role of beta oscillations in maintaining cognitive states, endogenous task representations, and top-down regulation (Engel and Fries, 2010; Spitzer and Haegens, 2017). Moreover, frontoparietal beta-band activity has been implicated in working memory and executive processing, where it is thought to support the maintenance and manipulation of task-relevant information across distributed cortical networks (Chang et al., 2023; Sattelberger et al., 2024). Although comparatively little literature has specifically addressed hemispheric beta asymmetry, the present findings suggest that lateralized beta activity may provide complementary information to conventional bandpower analyses and warrants further investigation.

Taken together, the asymmetry analyses complemented the bandpower results by revealing condition-dependent lateralization patterns. Whereas alpha asymmetry primarily differentiated the distraction condition, beta asymmetry emerged as the most consistent hemispheric marker across increasing cognitive demands, suggesting that hemispheric beta dynamics may provide complementary information beyond conventional regional bandpower analyses.

### 4.4 Functional connectivity

Contrary to our initial hypothesis, inferential analyses did not reveal statistically significant condition-related differences in functional connectivity after correction for multiple comparisons. This finding was consistent across both the network-based statistics and the edge-wise linear mixed-effects analyses. Nevertheless, the descriptive connectivity matrices exhibited frequency- and condition-specific spatial patterns that partially resembled previously reported connectivity distributions and may provide hypotheses for future studies.

Descriptively, theta-band connectivity appeared more spatially extensive during the time-pressure and combined conditions, whereas alpha-band connectivity showed comparatively reduced synchronization across frontal regions. Similar increases in frontoparietal theta synchronization and reductions in alpha synchronization have frequently been associated with cognitive control, attentional processing, and increasing task demands (Cavanagh and Frank, 2014; Dai et al., 2017; Muthukrishnan et al., 2020). However, because these patterns did not reach statistical significance in the present analyses, they should be regarded as qualitative observations rather than evidence of condition-dependent connectivity changes.

Several methodological factors may explain the absence of statistically significant connectivity effects despite the descriptive spatial patterns. ROI averaging increases robustness by reducing channel-level variability but may also obscure localized connectivity changes. Furthermore, connectivity measures such as the weighted phase lag index quantify interregional phase synchronization and generally exhibit greater inter- and intra-individual variability than local oscillatory power estimates while intentionally suppressing zero-lag interactions to reduce spurious connectivity (Vinck et al., 2011; Bastos and Schoffelen, 2016). Consequently, connectivity analyses often require larger sample sizes, longer recording durations, or higher spatial resolution to achieve statistical sensitivity comparable to regional bandpower analyses (Cohen, 2014; Siegel et al., 2012).

### 4.5 Global changes

Time pressure exhibited significant frontal-midline theta-band increases accompanied by widespread alpha-band decreases across frontal and parietotemporal regions. In contrast to distraction, alpha asymmetry no longer reached statistical significance, whereas beta asymmetry remained significant. Functional connectivity again did not reveal statistically significant condition-related effects, although the descriptive theta-band connectivity pattern was spatially most pronounced in this condition. Overall, the oscillatory profile is consistent with increased cognitive control and sustained attentional allocation under time pressure, accompanied by persistent hemispheric reorganization reflected in beta asymmetry.

The combined condition was characterized by left frontal theta-band increases, lateral central alpha-band decreases, and a localized right frontal beta-band increase. Beta asymmetry again was statistically significant, whereas alpha asymmetry exhibited its largest *t*-values despite not reaching statistical significance. As in the individual conditions, inferential connectivity analyses remained non-significant, although the descriptive connectivity matrices displayed a distinct spatial organization. Rather than representing a simple superposition of distraction and time pressure, the combined condition elicited a distinct oscillatory organization involving a distinct oscillatory organization combining features associated with attentional filtering and executive control.

### 4.6 Limitations

Several limitations should be considered when interpreting the present findings. First, the study included a limited sample size, which may limit the generalizability of the observed effects and reduce sensitivity to smaller neural modulations. In particular, recent studies have reported sex-specific neural and behavioral responses to cognitive demand and workload (Wriessnegger et al., 2024, 2025; Leitner and Wriessnegger, 2025). Although such effects may also be relevant in the present paradigm, a dedicated investigation of sex-dependent neural responses was beyond the scope of the current study and would require larger cohorts to ensure sufficient statistical power. Second, although the paradigm combined simultaneous auditory distraction and time pressure to increase ecological validity, the laboratory-based working memory paradigm should be regarded as an intermediate step toward more realistic real-life paradigms. Although the time-pressure manipulation increased subjective workload ratings, the study did not include condition-specific physiological stress measures. Consequently, the findings should primarily be interpreted in terms of increased cognitive demand and subjective workload rather than a comprehensive physiological stress response. Future work may extend the present framework by incorporating additional stress-sensitive EEG metrics, including spectral ratios, entropy-based measures, and fractal characteristics, to provide a more comprehensive characterization of cognitive and attentional demands. Although the auditory distraction recordings had been successfully employed in our previous studies, they were not independently validated with respect to their distracting properties. Future studies should evaluate whether standardized and perceptually validated auditory stimuli produce comparable neural and behavioral effects. Future work should additionally consider spectral parameterization approaches [e.g., specparam/FOOOF (Donoghue et al., 2020; Gerster et al., 2022)] to separate periodic oscillatory activity from the aperiodic 1/f component, thereby enabling a more refined interpretation of condition-dependent spectral changes.

## 5 Conclusion

The present study provides a multidimensional EEG characterization of distraction, cognitive demand induced by time pressure, and their combination during working memory performance. Across bandpower and hemispheric asymmetry analyses, different EEG patterns emerged for both manipulations, whereas functional connectivity analyses did not reveal significant inferential effects. Distraction was primarily characterized by widespread alpha suppression together with significant frontal and parietal delta-band increases, whereas time pressure elicited a theta-dominated pattern with enhanced frontal and parietal theta activity. The combined condition exhibited a distinct spatial pattern of oscillatory changes rather than a simple additive superposition of the isolated effects. Hemispheric asymmetry analyses complemented these findings by revealing condition-dependent lateralization patterns, with beta asymmetry showing the most consistent condition related lateralization pattern across the experimental conditions. Behaviorally, participants largely maintained response speed despite increased subjective workload, whereas the combined condition was associated with reduced accuracy, suggesting limits of compensation under simultaneous cognitive demands.

Together, these findings across behavioral performance, oscillatory activity, and hemispheric asymmetry contribute to a more comprehensive understanding of neural responses to concurrent cognitive demands and highlight the value of multidimensional EEG approaches for investigating realistic task environments. Although functional connectivity analyses did not identify significant condition-dependent network changes, they complement the multidimensional framework and motivate further investigation using larger datasets and higher spatial resolution. Future work should further investigate hemispheric asymmetry, beta-band dynamics, sex-specific neural responses, and spectral parametrization approaches, which may provide additional insights for neuroadaptive technologies aimed at distinguishing adaptive engagement from emerging cognitive overload.

## Data Availability

The raw data supporting the conclusions of this article will be made available by the authors, without undue reservation.
