# Supplementary material for: Neural signatures of auditory distraction and cognitive demand during working memory performance
Source: Front Neurosci. 2026 Sep 23;20:1919376. doi: 10.3389/fnins.2026.1919376 (PMC13642836; doi:10.3389/fnins.2026.1919376)
Supplement: Supplementary file 1 [file Table_1.pdf]

SUPPLEMENTARY DATA

Only electrodes with FDR-corrected $p < 0.050$ are shown.

Combined vs Baseline

Feature	Electrode	p_{FDR}	Estimate	95% CI
BP — Alpha	C3	7.59e-04	-0.068	[-0.101, -0.036]
	FC6	7.59e-04	-0.068	[-0.101, -0.035]
BP — Beta	F8	0.008	0.060	[0.028, 0.092]
BP — Theta	F3	0.008	0.073	[0.033, 0.113]
	T8	0.008	0.067	[0.029, 0.105]
	TP10	0.027	0.058	[0.020, 0.096]
Beta asymmetry	Fp1	7.46e-06	-0.052	[-0.072, -0.032]
	Fp2	7.46e-06	0.052	[0.032, 0.072]
	TP10	2.34e-05	-0.064	[-0.091, -0.037]
	TP9	2.34e-05	0.064	[0.037, 0.091]
	C3	0.003	0.058	[0.024, 0.091]
	C4	0.003	-0.058	[-0.091, -0.024]
	FC5	0.003	0.043	[0.018, 0.069]
	FC6	0.003	-0.043	[-0.069, -0.018]
	CP5	0.021	0.041	[0.011, 0.072]
	CP6	0.021	-0.041	[-0.072, -0.011]
	FT10	0.031	-0.035	[-0.063, -0.007]
	FT9	0.031	0.035	[0.007, 0.063]

Distraction vs Combined

Feature	Electrode	p_{FDR}	Estimate	95% CI
BP — Delta	FC2	0.001	0.077	[0.041, 0.114]
	Fz	0.039	0.058	[0.020, 0.096]
Beta asymmetry	T7	0.002	-0.055	[-0.084, -0.025]
	T8	0.002	0.055	[0.025, 0.084]
	TP10	0.002	0.052	[0.025, 0.078]
	TP9	0.002	-0.052	[-0.078, -0.025]

Distraction vs Baseline

Feature	Electrode	p_{FDR}	Estimate	95% CI
Alpha asymmetry	F7	0.008	0.054	[0.021, 0.086]
	F8	0.008	-0.054	[-0.086, -0.021]
	Fp1	0.008	0.051	[0.020, 0.083]

Feature	Electrode	p_{FDR}	Estimate	95% CI
	Fp2	0.008	-0.051	[-0.083, -0.020]
BP — Alpha	FC6	1.44e-05	-0.085	[-0.118, -0.052]
	CP5	0.003	-0.063	[-0.097, -0.029]
	T8	0.003	-0.060	[-0.092, -0.028]
	CP6	0.006	-0.058	[-0.092, -0.024]
	F4	0.006	-0.055	[-0.087, -0.022]
	C3	0.006	-0.054	[-0.087, -0.022]
	FC2	0.019	-0.050	[-0.084, -0.016]
	CP1	0.026	-0.043	[-0.074, -0.012]
	P3	0.044	-0.040	[-0.071, -0.009]
BP — Delta	CP5	0.030	0.058	[0.021, 0.095]
	FC2	0.030	0.059	[0.022, 0.096]
	P7	0.030	0.056	[0.019, 0.093]
	FC6	0.037	0.052	[0.016, 0.089]
	F4	0.044	0.052	[0.014, 0.089]
Beta asymmetry	Fp1	2.63e-07	-0.058	[-0.079, -0.038]
	Fp2	2.63e-07	0.058	[0.038, 0.079]
	CP5	0.006	0.052	[0.021, 0.082]
	CP6	0.006	-0.052	[-0.082, -0.021]
	C3	0.011	0.052	[0.018, 0.085]
	C4	0.011	-0.052	[-0.085, -0.018]
	O1	0.015	0.048	[0.015, 0.081]
	O2	0.015	-0.048	[-0.081, -0.015]

688 **Time pressure vs Combined**

Feature	Electrode	p_{FDR}	Estimate	95% CI
BP — Beta	F8	0.015	-0.057	[-0.089, -0.025]

689 **Time pressure vs Baseline**

Feature	Electrode	p_{FDR}	Estimate	95% CI
BP — Alpha	F3	0.014	-0.056	[-0.091, -0.022]
	F4	0.014	-0.056	[-0.089, -0.023]
	FC6	0.014	-0.051	[-0.084, -0.019]
	Fz	0.014	-0.058	[-0.092, -0.023]
	T8	0.014	-0.051	[-0.083, -0.019]
	CP5	0.015	-0.053	[-0.087, -0.018]
	CP1	0.015	-0.047	[-0.078, -0.016]
	C3	0.016	-0.048	[-0.080, -0.015]

Feature	Electrode	p_{FDR}	Estimate	95% CI
	CP6	0.016	-0.049	[-0.083, -0.015]
	TP9	0.036	-0.044	[-0.079, -0.010]
BP — Theta	Oz	3.96e-05	0.094	[0.056, 0.132]
	Fz	8.09e-05	0.087	[0.048, 0.125]
	O1	8.09e-05	0.085	[0.048, 0.122]
	O2	8.09e-05	0.086	[0.048, 0.124]
	Cz	1.83e-04	0.079	[0.042, 0.116]
	F3	5.67e-04	0.079	[0.039, 0.119]
	FC2	5.92e-04	0.073	[0.036, 0.111]
	FC1	9.12e-04	0.074	[0.035, 0.113]
	TP10	0.007	0.059	[0.022, 0.097]
	P8	0.009	0.059	[0.020, 0.097]
	P3	0.013	0.057	[0.018, 0.096]
	P7	0.018	0.053	[0.014, 0.091]
	T8	0.018	0.052	[0.014, 0.089]
	TP9	0.018	0.049	[0.013, 0.086]
Beta asymmetry	FC5	5.77e-04	0.054	[0.028, 0.079]
	FC6	5.77e-04	-0.054	[-0.079, -0.028]
	Fp1	0.029	-0.029	[-0.049, -0.009]
	Fp2	0.029	0.029	[0.009, 0.049]
	TP10	0.029	-0.038	[-0.065, -0.011]
	TP9	0.029	0.038	[0.011, 0.065]

690 **Time pressure vs Distraction**

Feature	Electrode	p_{FDR}	Estimate	95% CI
BP — Beta	F3	0.041	0.049	[0.019, 0.079]
BP — Delta	FC2	0.007	-0.066	[-0.103, -0.029]
	Fz	0.007	-0.069	[-0.107, -0.031]
	F4	0.018	-0.060	[-0.098, -0.023]
	F3	0.029	-0.054	[-0.091, -0.018]
BP — Theta	Fz	4.62e-06	0.103	[0.065, 0.142]
	O1	2.13e-04	0.081	[0.044, 0.118]
	Oz	2.13e-04	0.084	[0.046, 0.122]
	FC1	0.002	0.073	[0.034, 0.113]
	O2	0.002	0.072	[0.034, 0.110]
	P3	0.004	0.067	[0.028, 0.106]
	F7	0.006	0.065	[0.026, 0.104]
	FT9	0.024	0.055	[0.016, 0.094]
